# Protein adsorption through Chitosan–Alginate membranes for potential applications

**DOI:** 10.1186/s13065-016-0167-y

**Published:** 2016-04-30

**Authors:** Dennise A. Murguía-Flores, Jaime Bonilla-Ríos, Martha R. Canales-Fiscal, Antonio Sánchez-Fernández

**Affiliations:** Tecnologico de Monterrey, Campus Monterrey, Av. Eugenio Garza Sada Sur 2501, Tecnológico, 64849 Monterrey, Nuevo León Mexico

**Keywords:** Cellulose beads, ​Chitosan, Sodium alginate, Adsorption, Filtration, Membrane

## Abstract

**Background:**

Chitosan and Alginate were used as biopolymers to prepare membranes for protein adsorption. The network requires a cross-linker able to form bridges between polymeric chains. Viscopearl-mini^®^ (VM) was used as a support to synthesize them. Six different types of membranes were prepared using the main compounds of the matrix: VM, Chitosan of low and medium molecular weight, and Alginate.

**Results:**

Experiments were carried out to analyze the interactions within the matrix and improvements were found against porous cellulose beads. SEM characterization showed dispersion in the compounds. According to TGA, thermal behaviour remains similar for all compounds. Mechanical tests demonstrate the modulus of the composites increases for all samples, with major impact on materials containing VM. The adsorption capacity results showed that with the removal of globular protein, as the adsorbed amount increased, the adsorption percentage of Myoglobin from Horse Heart (MHH) decreased. Molecular electrostatic potential studies of Chitosan–Alginate have been performed by density functional theory (DFT) and ONIOM calculations (Our own N-layered integrated molecular orbital and molecular mechanics) which model large molecules by defining two or three layers within the structure that are treated at different levels of accuracy, at B3LYP/6-31G(d) and PM6/6-31G(d) level of theory, using PCM (polarizable continuum model) solvation model.

**Conclusions:**

Finally, Viscopearl-mini^®^ acts as a suitable support on the matrix for the synthesis of Chitosan–Alginate membranes instead of cross-linkers usage. Therefore, it suggests that it is a promise material for potential applications, such as: biomedical, wastewater treatment, among others.

**Graphical abstract:**
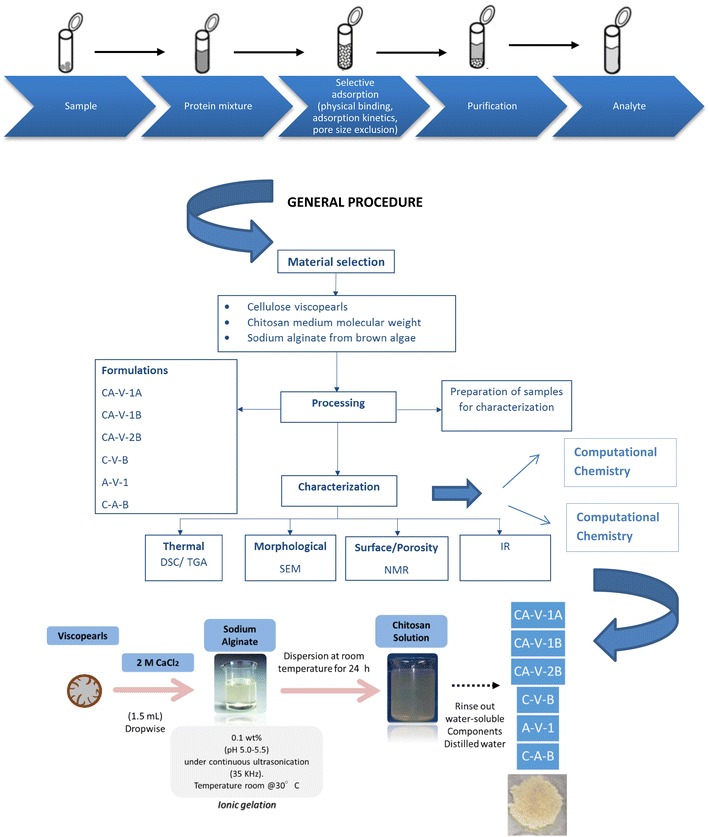
Chitosan, Alginate, and Cellulose beads-based membranes for protein adsorption. Special attention was given for preparation, charaterization, adsorption capacity, and molecular electrostatic potential studies calculation. Viscopearl-mini^®^ gives support on the matrix of Chitosan–Alginate membranes instead of cross-linkers usage

## Background

Polymeric materials constitute a fast-growing area within the global economy, confirmed by the continuous and dynamic production of plastics [1]. Because of the limited source of mineral raw materials and environmental protection, new sources of raw materials can be retaken to produce polymers [2]. The Chitosan, Alginate, and Cellulose biopolymers may have the potential to be used as low-cost raw materials since they represent widely available and environmentally friendly resources [2] that seem attractive for the use, not only in medicine and tissue engineering (TE) [3], among others. Biodegradable polymers produced from renewable resources represent plastics that may contribute to the enhancement of natural environment protection [4–7]. Porous matrices from biomaterials [8] are used in the generation of porous matrices which include collagen [9], gelatin [10] silk [11], alginate [12], and Chitosan [11]. Alginate is a natural linear polysaccharide copolymer produced by brown algae, and bacteria. It is widely used because of its ability to form strong thermo-resistant gels, non-toxicity, biodegradability, high biocompatibility [11], and widely used in medical applications [13] such as tissue TE [14]. Cellulose is mostly used in the paper, textile and medical industry [15]. Chitosan has excellent chemical properties such as, adsorption [16]; due to the reactive number of the available hydroxyl groups, reactive amino groups, and a flexible polymer chain structure [17, 18]. However, used as an adsorbent brings some drawbacks such as low surface area or porosity, high cost, and poor chemical and mechanical properties [19, 20]. Physical or chemical modifications have been studied, such as: copolymerization, grafting, or cross-linking processes [2, 21–24].

The conjunction of different biopolymers is an extremely attractive, inexpensive and advantageous method to obtain new structural adsorbent materials [25].

Materials such as fly ash, silica gel, zeolites, lignin, seaweed, wool wastes, agricultural wastes, clay materials, and sugar cane bagasse, among others, have been extensively used for protein removal, due to their sorption sites [15].

Cellulose-based composite hydrogels blended with various biopolymers can create novel materials for special applications [26–32]. The widespread applications of porous materials is not limited as adsorbents for small active molecules. Various polysaccharide hydrogels have been employed for the entrapment of enzymes [33–40]. Furthermore, specific pore structures and tunable morphology allow the construction of affinity probes for various macromolecules [40]. The usage of porous adsorbents for selective and fast separation of phosphorylated proteins and peptides (β-caseine) [41]; real samples of human serum [41], and human urine have been captured with Fe_3_O_4_ magnetic micro-spheres coated with TiO_2_-incorporated mesoporous silica [42, 43] have been recently developed.

On the other hand, microspheres favourably affect mechanical properties of polymers such as modulus of elasticity, tensile strength, hardness, and abrasion resistance [3]. These materials could be reused several times; therefore, they become important in terms of their valuable and unique functional properties. Compounds obtained from mechanical recycling of materials can be completely profitable due to lower costs of biodegradable materials and the possibility to avoid a considerable amount of industrial waste [3].

In the study of adsorbents the determination of adsorption capacity is fundamental. In this case, DFT (density functional theory) calculations represent the most suitable method for investigation involving systems with large molecules such as porphyrins [44–47]. Becke combined with the Lee–Yang–Parr correlation density functional method (B3LYP) is utilized due to highest theoretical and experimental correlation data [48, 49]. Researchers have employed the gradient-corrected DFT (6-31G basis set) on heavy atoms [49, 50].

To our knowledge, the studies focused on Myoglobin from horse heart (MHH) adsorption performance CA-cellulose viscopearls membranes at different temperatures, and evaluating equilibrium, thermodynamic, and kinetic parameters based on temperature of the system, are very limited.

The objective of this study is to determine and compare the adsorption performances of the CA-cellulose viscopearl membranes in the adsorption removal process of MHH from aqueous solutions at different temperatures in view of equilibrium, kinetic, and thermodynamic studies, using both Langmuir equilibrium constant (*K*_*L*_) and solute distribution coefficient (*K*_*d*_) [51]. This, in turn, should stimulate research in the field of investigation of such reinforced biomaterials.

The above-mentioned issues inspired authors to undertake research works aimed at comparison of changes in: (a) adsorption process [mean free adsorption Energy (*E*_*fe*_)], kinetic diffusion properties [the intraparticle diffusion coefficient (*D*_*p*_) and film diffusion coefficient (*D*_*f*_)], and thermodynamic parameters; (b) tensile strength, (c) tensile strain at break, (d) flexural strength, (g) thermal properties [thermogravimetric analysis (TGA)], (h) structural properties of samples [Fourier transform infrared spectroscopy (FT-IR)], and (i) surface free energy (solid-state carbon-13 nuclear magnetic resonance (solid state ^13^C-NMR) spectroscopy [52]), and (j) mechanism of interaction, deformation of compounds, and adsorption energies [ONIOM and molecular dynamics (MD)]. The results are offered in the present paper.

## Results and discussion

### Adsorption experiments

**Fig. 1:**
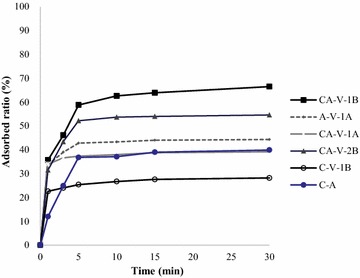
Effect of contact time on the equilibrium adsorption capacity of different initial concentration of Myoglobin at 30 °C, CA-cellulose viscopearl membrane dose of 0.5 g/L at 1000 mg/L

**Fig. 2:**
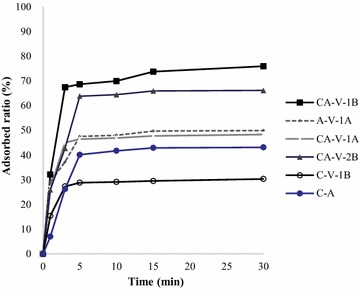
Effect of contact time on the equilibrium adsorption capacity of different initial concentration of Myoglobin at 30 °C, CA-cellulose viscopearl membrane dose of 0.5 g/L at 500 mg/L

Contact time is a parameter that determines the rate of Myoglobin removal; the results of initial Myoglobin concentrations for all samples are shown in Figs. 1 and 2. The data show that the adsorption capacity of Myoglobin increases with the increase of MHH concentration. The adsorption process for Myoglobin has two stages. The fastest rate of adsorption was found after the first 10 min and the equilibrium was attained in about 30 min. The q_e_ value and adsorption capacity are higher at the beginning due to the large surface area of adsorbents available for adsorption of Myoglobin.

Figures 1 and 2 also show that an increase in initial MHH concentration decreases the adsorbed ratio. This can be attributed to the increase in the number of MHH molecules competing for available binding sites on the CA-cellulose viscopearls membranes. Thus, the available active sites of the CA-cellulose viscopearl membranes become saturated at higher concentration of MHH [53, 54].

Thermodynamic parameters, such as change in Gibbs free energy, were determined using the classic Van’t Hoff equation:

1 $$\Delta G^{0} = \, {-}RT\;{ \ln }\;K$$

where *ΔG*^*0*^ is the standard free energy change (kJ/mol), *T* is the absolute temperature, *R* is gas constant (J/mol K), and *K* is an equilibrium constant obtained by multiplying the Langmuir constants *q*_*m*_ and *K*_*L*_ [55]. The value of *ΔG*^*0*^ is used to determine the nature of the adsorption process. The determined *ΔG*^*0*^ is −4.1 kJ/mol. The *ΔG*^*0*^ for physisorption ranges from −20 kJ/mol to 0 kJ/mol and for chemisorption, it ranges from −80 kJ/mol to −400 kJ/mol [56, 57]. The values of *ΔG*^*0*^ indicated that the adsorption can be designated as spontaneous physisorption. The *ΔG*^*0*^ for hydrogen bonding and dipole force are 2–40 kJ/mol and 2–29 kJ/mol, respectively [58–60]. The results suggest that the interaction between the adsorbent and the adsorbate is hydrogen bonding with a weak attractive force.

It was important to measure the protein adsorption capacity of the material as well as its capacity to retain the adsorbed compound into polymer matrix so that it could be reusable. In order to determine MMH protein desorption of the membrane, a new compound was prepared. From the CA-V-1A compound, which is the one with the highest protein adsorption capacity, the same formulation was used to synthesize compound P-1000 in which a solution of 1000 ppm is added to MHH during preparation. This occurs after incorporating the Alginate solution and allowing the sample to dry (see “Preparation of Chitosan Alginate (CA)-cellulose viscopearl membranes” section).

After the synthesis of compound P-1000, the sample N-P was encoded and subjected to seven rinses with distilled water at room temperature. These experiments for washing the sample were carried out with 10 mL of MHH; the solution passed through a Hirsch funnel containing the samples by applying vacuum pressure. P-1000 samples of 0.5 g were tested with 1000 mg/L of MHH solutions whose concentration corresponds to 1000 ppm.

### Adsorption equilibrium and calculation of mean free sorption energy

In this investigation, the most frequently used equations, Langmuir and Freundlich isotherm models, were used to analyze the isotherm data for the purpose of optimizing the design of an adsorption system. It is also an important step to establish the suitable correlation for equilibrium conditions.

The corresponding mean free adsorption Energy (*E*_*fe*_) was calculated to interpret the mechanism of MHH removal; meanwhile, the intraparticle diffusion coefficient (*D*_*p*_) and film diffusion coefficient (*D*_*f*_) were calculated separately to describe the kinetic diffusion process of MHH adsorption. Also, thermodynamic parameters like *ΔG*^*0*^, *ΔH*^*0*^, and *ΔS*^*0*^ were respectively calculated using both Langmuir equilibrium constant (*K*_*L*_) and solute distribution coefficient (*K*_*d*_), in order to compare the different thermodynamic calculation methods [51].

This investigation presents a combined study of ONIOM and molecular dynamics (MD) aimed to understand the mechanisms of interaction and deformation of analyzed compounds. Likewise, adsorption analysis is performed considering the most stable structure of the system at geometrical parameters changes and adsorption energies.

Equilibrium data, known as adsorption isotherms, are basic parameters for the design of adsorption systems. In order to calculate the adsorption capacity of Chitosan–Alginate membranes, the experimental data were fitted to the Linearized Langmuir isotherm and Linearized Freundlich isotherm, Eqs. (2) and (3), respectively [61, 62]:

Linearized Langmuir isotherm is given by the following equation:

2 $$1/q_{\text{e}} = { 1}/\left( {q_{\text{m}} K_{\text{L}} C_{\text{e}} } \right) \, + { 1}/q_{\text{m}}$$

where *q*_m_ is the Langmuir constant relating to complete coverage (mg/g) and *K*_L_ is the Langmuir energy constant which indicates adsorptivity of the solute. This empirical model is based on the following assumptions involving homogeneous adsorption situation. The Langmuir model is typically considered to be suitable for fitting the adsorption type onto organic adsorbents; however, it is restricted to some harsh terms: it assumes that a monolayer adsorption takes place on a homogeneous surface of adsorbent, and that there is no interaction between neighbouring adsorbed species [63, 64].

The linear form of Freundlich isotherm is given by the following equation:

3 $${ \log }q_{\text{e}} = \, \left( { 1/n} \right){ \log }C_{\text{e}} + { \log }K_{\text{F}}$$

where *n* is the Freundlich isotherm constant related to adsorption intensity and *K*_F_ is the Freundlich isotherm constant related to adsorption capacity (mg/g)(L/mg)^1/*n*^.

**Fig. 3:**
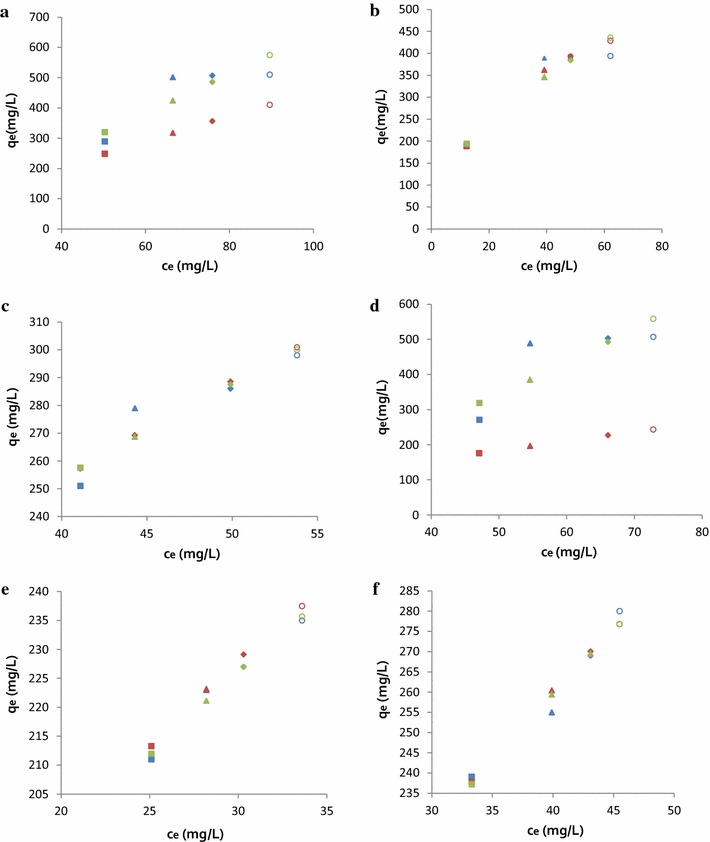
Adsorption isotherm of the adsorption of MHH on CA-cellulose viscopearls samples: **a** CA-V-1B; **b** CA-V-1A; **c** A-V-1A; **d** CA-V-2B; **e** C-V-1B; **f** CA 2000, 1000, 500, 250 mg L^−1^, stirred slowly, adsorbent 0.5 g, adsorption time 30 min (303 K). Also, the lines include linear fitting curves with Langmuir and Freundlich model, and experimental results (identified *colors*)

Table 1 summarizes the results of adsorption capacity for all samples and, along Fig. 3, shows that the Freundlich model fits slightly better with the decrease in concentration (from 250 to 2000 ppm) at 303 K when comparing the R_2_ values (from Excel, Display R-squared value on chart) with the Langmuir model. The different types of membrane formulation in contact with a higher concentration of MHH adsorption solution showed lower interaction in the active adsorption sites. In addition, the increase in the concentration can widen the pores of resin particles and can increase the activity of sorption sites.

**Table 1:** Freundlich and Langmuir isotherm parameter for adsorption capacity (303 K)

Compound	Cellulose viscopearls (gr)		Alginate	Chitosan		Code name
	0.33 wt%	0.5 wt%	0.16 wt%	LMM 0.42 wt%	MMW	
1	×		×		×	CA-V-1B
2	×		×			A-V
3	×		×	×		CA-V-1A
4		×	×		×	CA-V-2B
5	×				×	C-V-1B
6			×		×	C-A

First, the sorption takes place at specific homogeneous sites within the adsorbent. Second, no further sorption can take place at that site once a MHH molecule occupies it. Third, the adsorption capacity of the adsorbent is finite. Fourth, the size and shape of all sites are identical and energetically equivalent [63]. The Freundlich model is suitable for a highly heterogeneous surface composed of different classes of adsorption sites. This model has two main assumptions [63]: first, with the increase of surface coverage of adsorbent, the binding strength gradually decreases. Second, the adsorption energies of active sites on the surface of adsorbent are different.

Fitting the data with the Langmuir and Freundlich equations resulted in high correlation coefficients, varying from 0.99 to 1.00. This indicates that the Chitosan–Alginate membrane surfaces are homogeneous and coverage of MHH on the outer surface of samples is a monolayer adsorption [63, 64].

### Adsorption kinetics and calculation of activation energy

Figures 1 and 2 (see “Adsorption experiments” section) showed the effects of MHH initial concentration at 303 K on the CA-cellulose viscopearl sample. It can be observed that the variation of initial concentration of adsorption solution (500 and 1000 ppm) affected the rate of adsorption at initial period. This is due to the increase of initial concentration of adsorption solution and the MHH adsorption on each CA-cellulose viscopearl samples which gradually slowed down as concentration of adsorption solution increased; for each experiment the equilibrium was reached after 30 min. Besides the difference of concentration gradient, the interaction forces between solute and adsorbent become stronger than those between the solute and the solvent, leading to the fast adsorption at the initial stage [65]. As time passed, the sorption rate decreased, and temperature variation influencing the final adsorption capacity is not significant at the later equilibrium stage.

### Diffusion mechanism study

Three major rate limiting steps involving the kinetic diffusion mechanism are generally cited [66]: (a) film diffusion; (b) intraparticle diffusion; (c) interior surface diffusion; (d) adsorption or ion exchange on the pore surface. The intraparticle diffusion model (Weber–Morris model) is applied to analyze the empirically found functional relationship (q_t_ versus t_1/2_) [67].

Weber–Morris model:

4 $$q_{t} = k_{id} t^{1/2} + C_{i}$$

where *k*_*id*_ (*k*_*id*1_, *k*_*id*2_, and *k*_*id*3_) is defined as the intraparticle diffusion rate constant (mg mL^−1^ min^−1/2^), *k*_*id*1_ corresponds to the constant of the first stage involving external surface adsorption, *k*_*id*2_ is the constant of the second stage involving gradual adsorption, *k*_*id*3_ is shown as the constant of the third stage involving final equilibrium stage, and *C*_*i*_ represents the intercept reflecting the thickness of boundary layer.

According to the theory behind Weber–Morris model, the plot of *q*_*t*_ versus t_1/2_ should be linear when adsorption complies with the intraparticle diffusion mechanism and the intraparticle diffusion should be the only rate-determining step if the line passes through the origin. Otherwise, if the plots are multilinear, there are two or more rate-limiting steps involving in the adsorption process [68].

**Fig. 4:**
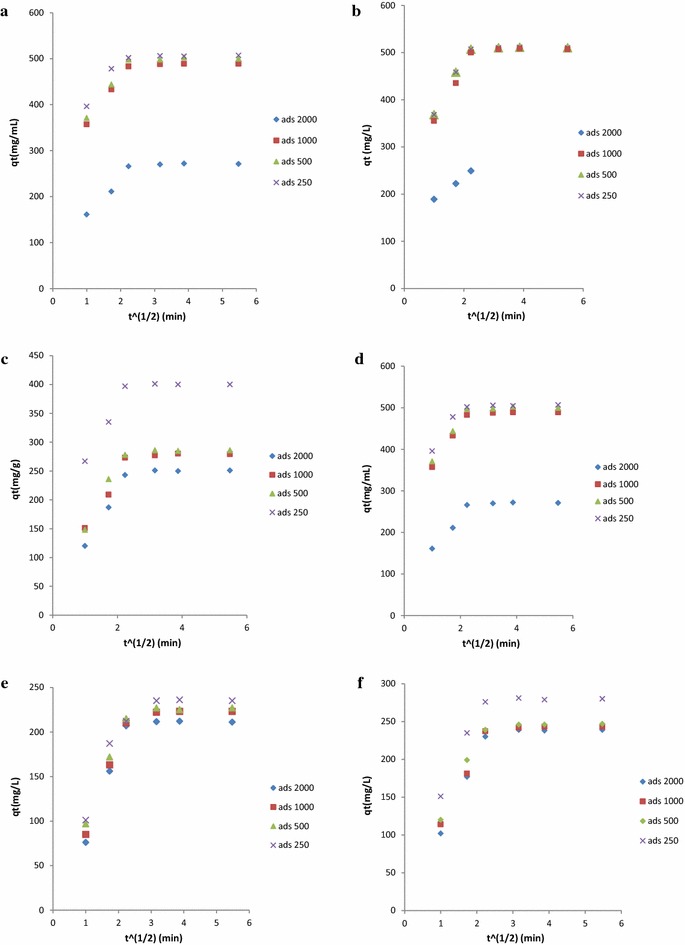
Plot of Weber–Morris intraparticle diffusion model for MHH adsorption on CA-cellulose viscopearl samples at T = 303 K; k_id1_, the first stage diffusion rate constant; k_id2_, the second stage diffusion rate constant; k_id3_, the third stage diffusion rate constant. On CA-cellulose viscopearls samples: **a** CA-V-1A; **b** CA-V-1B; **c** A-V-1A; **d** CA-V-2B; **e** C-V-1B; **f** CA. Concentration solution from 250 to 2000 ppm, manual stirring, adsorbent 0.5 g, temperature of 303 K

The values of *k*_*id*1_, *k*_*id*2_, *k*_*id*3_, and *C*_1_, *C*_2_, $C_{3 }$ for MHH adsorption at temperatures of 303 K are listed in Table 3. Figure 4 of *q*_*t*_ versus t_1/2_ showed that the MHH adsorption process was not linear over the entire time range and that adsorption was controlled by three different stages [69]: (1) instantaneous adsorption stage due to the external mass transfer; (2) intraparticle diffusion controlled gradual adsorption stage; and (3) final equilibrium stage due to the extremely low MHH concentration in the solution. For the above three stages, the second and third stage involved the intraparticle diffusion process. Figure 4 illustrated that intraparticle diffusion was not the rate controlling mechanism for all lines of stages 2 and 3 without passing through the origin. Moreover, the $k_{id1}$ values of the first portion for different temperature mg mL^−1^ min^−1/2^, respectively, were greater than *k*_*id*2_ and *k*_*id*3_ (Table 2). This indicated that external surface adsorption was faster compared with the intraparticle diffusion. The results further proved intraparticle diffusion was involved in the adsorption process but was not the only rate-limiting step throughout the adsorption process. Namely, other mechanisms (boundary layer diffusion or film diffusion) might contribute to the rate-determining step. The intraparticle diffusion coefficients *D*_*p*_ (m^2^ s^−1^) and film diffusion coefficients *D*_*f*_ (m^2^ s^−1^) have also been calculated to confirm the above results.

**Table 2:** Freundlich and Langmuir isotherm parameter for adsorption capacity intraparticle diffusion model parameters for the adsorption of MHH on CA-cellulose viscopearls at 1000 ppm of initial concentration of adsorption solution

	CA-V-1A	CA-V-1B	A-V-1A	CA-V-2B	C-V-1B	CA
K_L_ (L·mg^−1^)	0.036	0.005	0.015	0.006	0.059	0.027
q_m_ (mg·mL^−1^)	625	909.09	666.7	833.3	357.1	500
*R* ^*2*^	0.99	0.86	0.87	0.71	0.99	0.96
K_F_ (L·mg^−1^)·(L·mg^−1^)^1/n^	55.29	2.97	31.3	2.26	65.7	41.9
*N*	2.00	0.84	1.76	0.78	2.75	2.02
*1/n*	0.046	1.19	0.57	1.29	0.363	0.495
*R* ^*2*^	0.94	0.77	0.87	0.67	0.98	0.97

Intraparticle diffusion coefficient:

5 $$D_{p} = \frac{{0.03R_{p}^{2} }}{{t_{1/2} }}$$

Film diffusion coefficient:

6 $$D_{f} = \frac{{0.23R_{p} \varepsilon C_{s} }}{{t_{1/2} C_{L} }}$$

The average diameter of MHH particle was determined [70]. Then, the values of *D*_*p*_ and *D*_*f*_ were calculated under the given conditions explained below. *R*_*p*_ (m) is the average radius of the adsorbent particles, *ε* is the film thickness (10^−5^ m) [70] and *C*_*s*_ and *C*_*L*_ are the concentration of adsorbate in solid and liquid phase, respectively. Debnath et al. [70] assumed that the intraparticle diffusion will be the rate-limiting step if the calculated intraparticle diffusion coefficient (*D*_*p*_) value is in the range 10^−15^–10^−18^ m^2^ s^−1^. For the calculated film diffusion coefficient (*D*_*f*_) value ranging from 10^−10^ to 10^−12^ m^2^ s^−1^ the rate-limiting step is controlled by film diffusion. In this study, the calculated *D*_*p*_ values ranged from 1.81 10^−12^ to 11.2·10^−12^ m^2^ s^−1^, and the calculated values of *D*_*f*_ were found to be in the order of 10^−11^ m^2^ s^−1^.

Intraparticle diffusion coefficient (*D*_*p*_) and the film diffusion coefficient (*D*_*f*_) of adsorption process at 303 K at 1000 ppm and for CA-V-1B is Rp/m 1.8 × 10^−4^, the value for *t*_1/2_/*s* corresponds to 335.98, *D*_*p*_ (m^2^ s_−1_) is 2.56·10^−12^, and* D*_*f*_ (m^2^ s_−1_) calculated as 3.89 × 10^−11^.

Adsorption, the value of t_1/2_ is calculated by using the following equation [68]:

7 $$t_{1/2} = \frac{1}{{k_{2} q_{e} }}$$

### Characterization techniques

#### Thermal analysis

Measurements were carried out in a thermogravimetric-analyzer (TGA) from TA Instruments (STD Q600, New Castle, DE, USA).

**Fig. 5:**
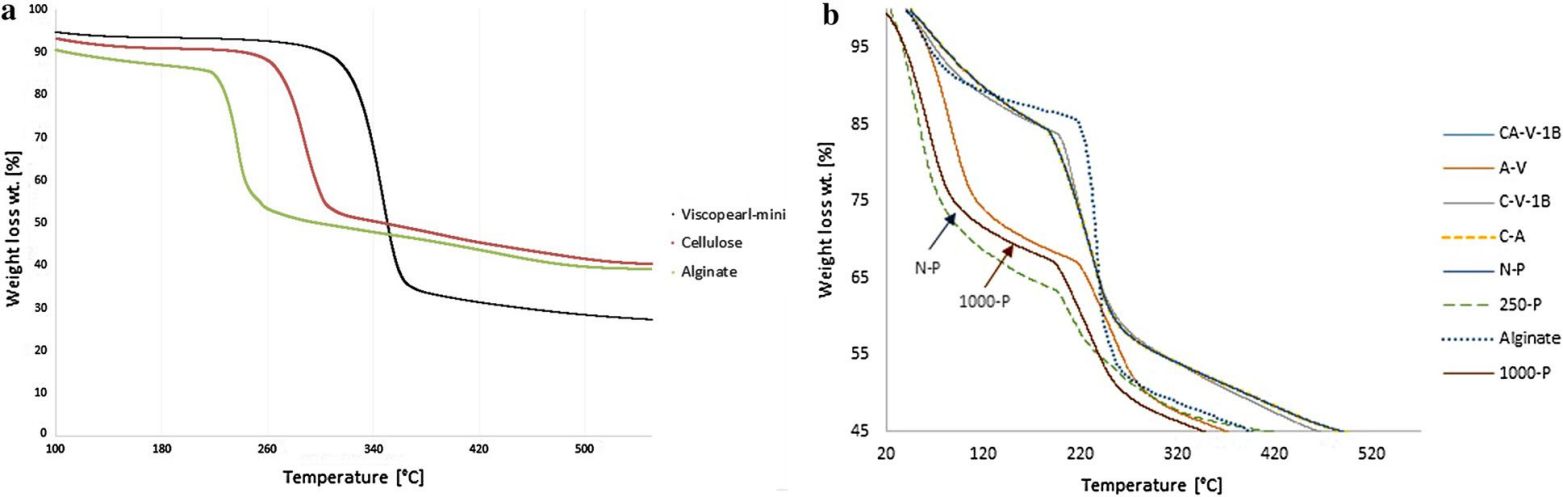
**a** Weight loss of Viscopearl-mini ^®^, weight loss of cellulose, weight loss of alginate; **b** weight of loss of CA-cellulose viscopearl membrane samples

TGA curves for the samples in nitrogen are shown in Fig. 5. The most notorious change in weight loss is presented in the range of 300–400 °C, although significant loss in mass starts around 400 °C. The range of temperature reveals that porous cellulose beads start degrading first. In the second and third stage it can be observed that the weight-loss percentage remain similar for the sample. The range 400–600 °C confirms that the lower degradation rate belongs to the functionalized porous cellulose beads. CA-cellulose viscopearl membranes containing Viscopearl-mini^®^ can be observed to be more stable.

#### IR

The IR spectra were carried out in an infrared spectrophotometer Thermo Nicolet^®^ model 6700 FTIR and using the attenuated total reflectance complement with diamond crystal. In order to analyze the data obtained, Omnic 7.3 software was used. The spectra were acquired in a range between 4000 and 400 cm^−1^ with a resolution of 4 cm^−1^ and 40 scans per analysis. A reference without the sample was registered before each analysis.

**Fig. 6:**
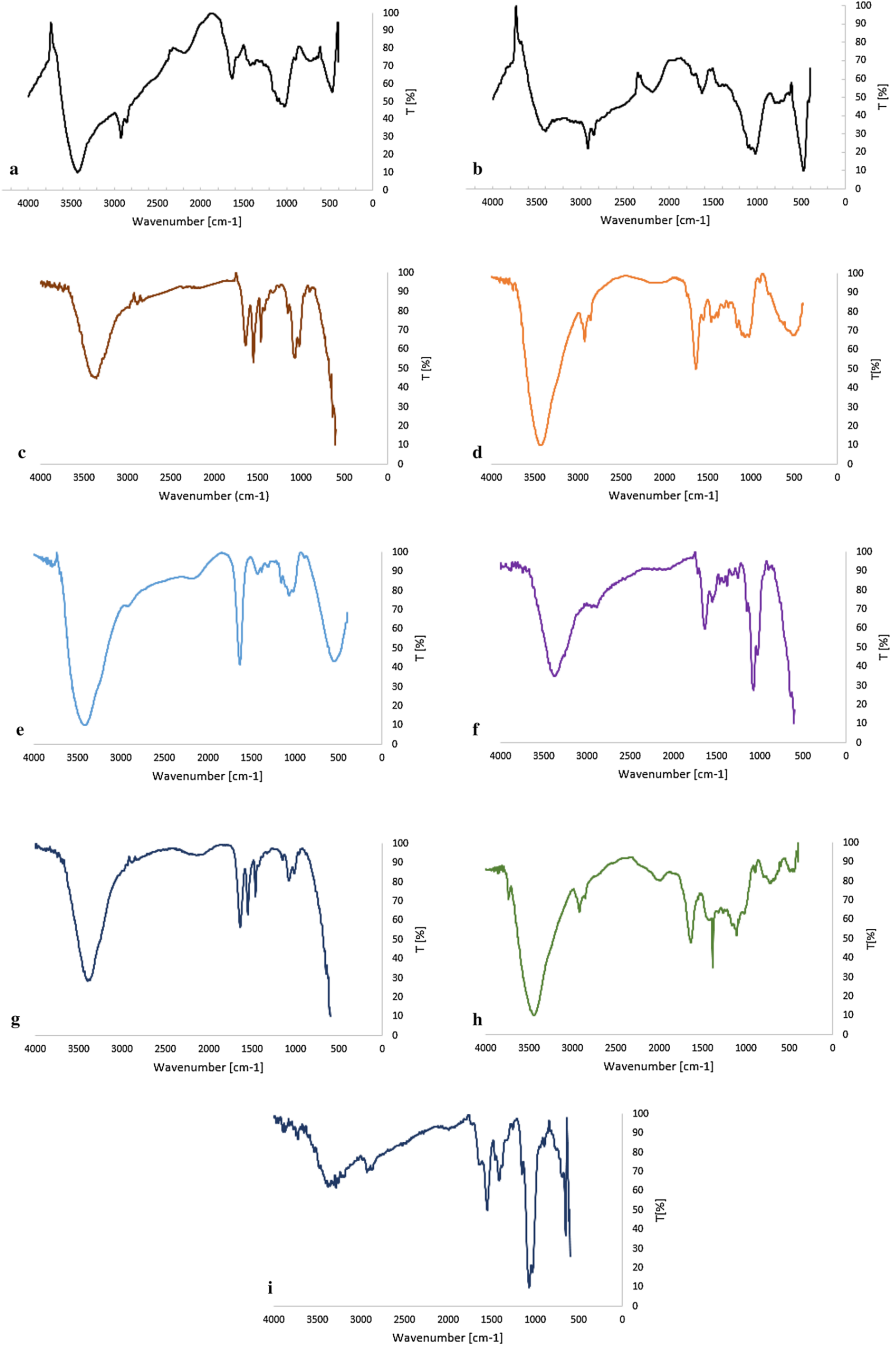
FTIR images of **a** CA-V-1A; **b** CA-V-1B; **c** C-V-1B; **d** CA-V-2B; **e** A-V; **f** C-A; **g** P-250; **h** P-1000; **i** N-P

Figure 6 depicts the FTIR spectrums of CA-V-1A, CA-V-1B, and Viscopearl-mini^®^. The peaks centered at 2850 and 2920 ῡ (cm^−1^) are due to C–H str (C–H stretching) and 1450 cm^−1^ for C–H bend (C–H bending). The bands at 1100 and 1000 cm^−1^ can be assigned to C–O from symmetric and incomplete network, respectively. Moreover, the peak at 3400 cm^−1^ suggests presence of hydroxyl groups in the blend (Cellulose, Alginate, Chitosan) and the intermolecular interactions with C=O groups. The absorption peak at 1650 cm^−1^ is characteristic of the carbonyl of the carboxylate and carboxylic acid.

IR bands characteristic of cellulose are distinguished: a broad hydrogen-bound O–H str band of the around 3400 cm^−1^, the C=O stretching band around 1650 cm^−1^ and the mixed C–O str and O–H str bands in the 1150–1350 cm^−1^ region, which suggest interactions between the cellulose components. These findings could indicate that Viscopearl-mini^®^ is esterified.

#### NMR

Solid-State ^13^C NMR spectroscopy is intrinsically a powerful and versatile tool for revealing the internal structure, composition, interface, and componential dynamics of polysaccharides. Therefore, to determine some structural differences related with the molecular mass of Chitosan, the samples CA-V-1A and CA-V-1B were analyzed by solid state ^13^C-NMR spectroscopy with an 11.7 Tesla Bruker Avance III equipment. Each sample was tested using cross-polarization (CP) and magic-angle spinning (MAS) with a rate of 125 MHz. A 4 mm inner diameter rotor with a spinning rate of 7 kHz was used. All ^13^C spectra were referenced to glycine (176.03 ppm, carbonyl, ^13^C).

**Fig. 7:**
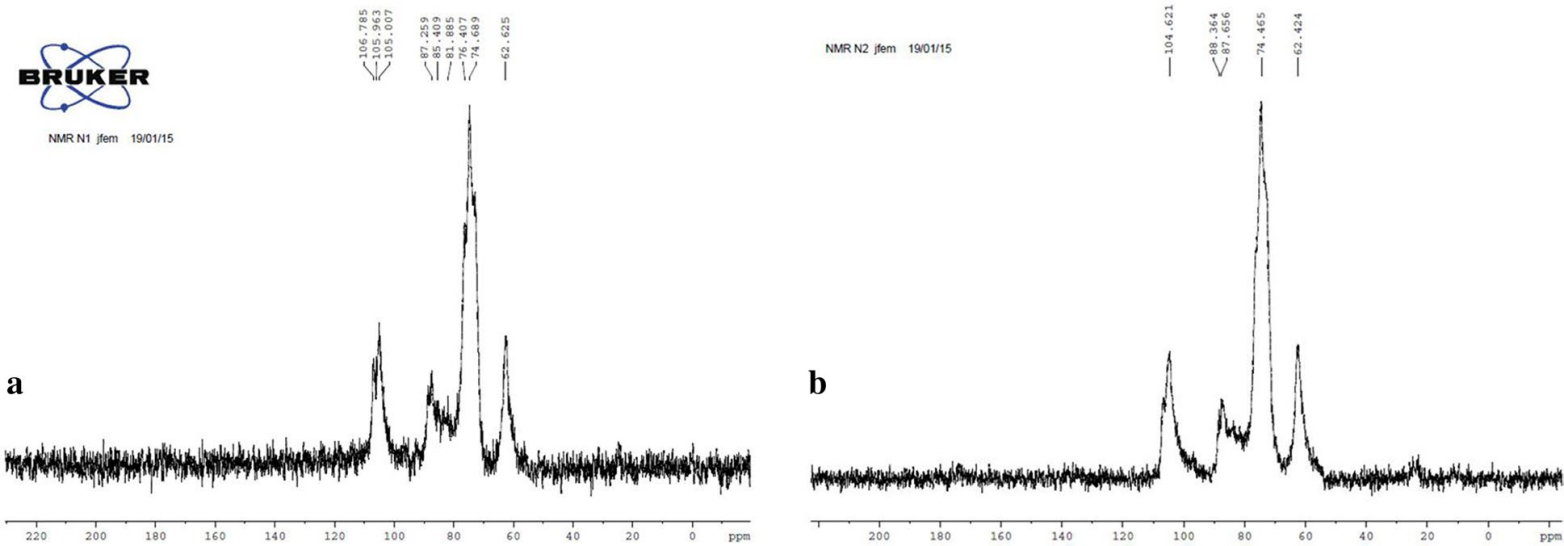
NMR images for images of **a** CA-V-1A; **b** CA-V-1B

Solid-state NMR (SSNMR) spectroscopy is a nondestructive and powerful technique for studying the multiscale structure, interfacial interaction, and dynamics of multiphase polymers at lengths ranging from the atomic level to approximately 100 nm [71]. A novel solid-state NMR approach based on ^1^H spin diffusion with X-nucleus (^13^C, ^31^P, ^15^N) detection was also proposed for investigation of the nanostructure of membrane proteins [72]. Figure 7 shows ^13^C CP-MAS NMR spectra of the blends CA-V-1A and CA-V-1B, showing the animatic carbons centered at 101 ppm and the ring carbons in the range of 60–90 ppm of Alginate, Cellulose and Chitosan.

#### SEM

In order to observe the particles dispersion on different prepared materials, SEM images were taken using a SEM-FEI Nova NanoSEM 200 (Hillsboro, TX, USA) microscope with an acceleration voltage of 10 kV and secondary electron detector under vacuum was used to characterize the morphology of the CA-cellulose viscopearls with protein immerse in the blending of CA-cellulose viscopearls formulation for their comparison. The Energy-dispersive X-ray spectroscopy (EDS) elemental analysis was carried out with an INCA-x-sight.

Scanning electron microscopy (SEM) analyses were conducted on cryofractured CA-cellulose viscopearl samples in order to investigate the dispersion of porous cellulose beads and interfacial features in membranes. This analysis is discarded only for the A-V compound because it was not possible to prepare the film.

**Fig. 8:**
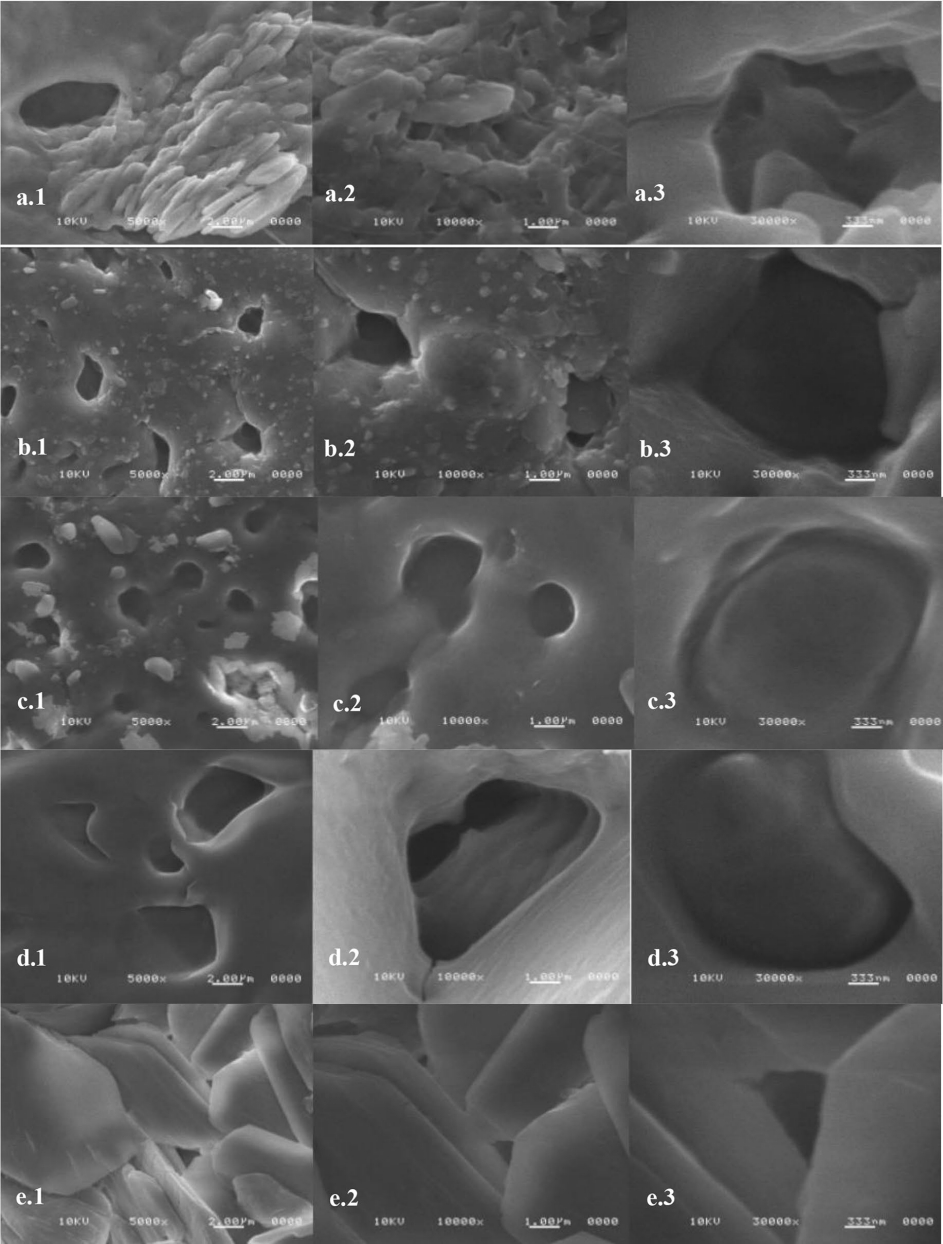
SEM images of **a** CA-V-1B; **b** CA-V-1A; **c** CA-V-2B; **d** C-V-1B; **e** C-A. From (**a**)–(**e**) images were taken: (1) ×5000, (2) ×10,000, (3) ×30,000

**Fig. 9:**
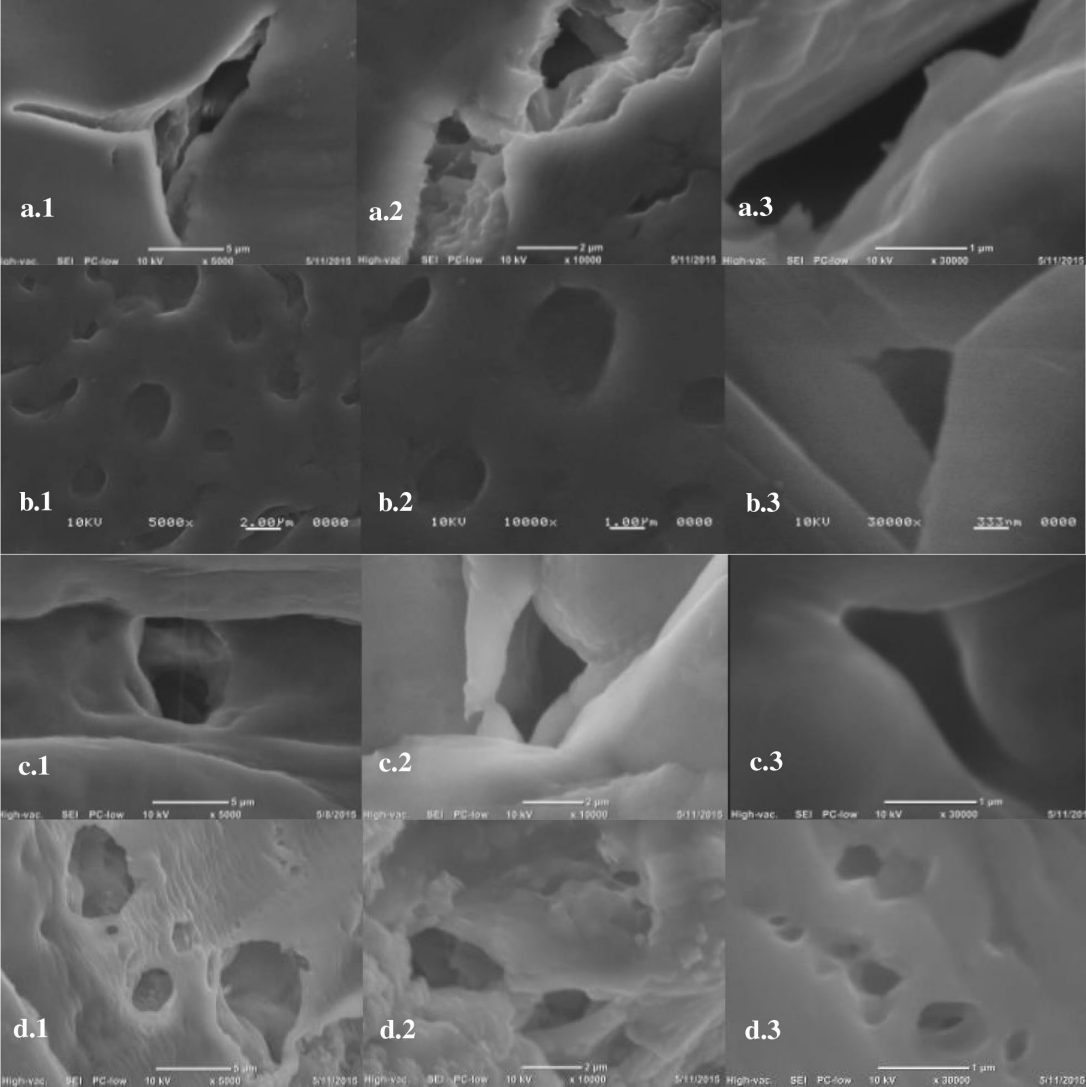
SEM images of **a** P; **b** N-P; **c** P-250; **d** P-2000. From (**a**)–(**c**) images were taken: (1) 5000×, (2) 10,000×, (3) 30,000×

SEM images of CA-cellulose, in a diameter range of 0.19–9.61 m, are shown in Fig. 8. Micrographs show that CA-V-1B (Fig. 8a), CA-V-1A (Fig. 8b), CA-V-2B (Fig. 8c), C-V-1B (Fig. 8d), C-A (Fig. 8e), P (Fig. 9a), N-P (Fig. 9b) have significant structural changes, showing particles and clusters formed and micrometric pores, differences in pore distribution, shape and size of cavities.

In order to observe the effect of MHH protein incorporation, P-250 (Fig. 9c), and P-2000 (Fig. 9d) samples were obtained. Those formulations were subjected to the same preparation as P-1000 (see “Thermal analysis” section). The results explain the difference of an increasing and decreasing MHH concentration.

SEM images showed porosity in the surface of CA-viscopearl membranes. A change in pore size can be observed which is assumed to be randomly distributed on the sample surface (see Table 3). Pore size of CA-V-1A was in the range of 0.19–0.5 m in the sample and more cavities were exposed to the surface. However, when compared to the others, the pore size of samples CA-V-1B, C-V-1B with CA-V-1A were larger, fewer, not round and had a different distribution of the cavities on the surface; therefore, they had lesser surface area than the others. This may explain the higher protein sorption capacity of the CA-V-1A. Likewise, a round shape and smaller pore size can be observed in C-V-1B sample. Due to lack of VM in the preparation of C-A membrane, a rough and non-porous surface was observed (Fig. 8e). SEM images for CA-V-2B suggest that the increase of VM incorporation resulted in an increasing of porosity; pore size was in the range of 0.75–2.85 m, and round shapes were observed. Figure 9a, which corresponds to P-1000 sample, showed a smooth surface, homogenous pore distribution, and smaller cavities formation compared to CA-V-1A where the difference could be attributed to the addition of protein. In the same sample, Fig. 9a 1 and 3 suggested a difference on their surface, pore size, and porosity dispersion according to the area where the micrograph was taken. Figure 9b) corresponds to N-P sample, in which pores are observed after washing out MMH protein from the P-1000 sample. Cavities of N-P sample appeared larger than P-1000; it could be concluded that MMH came out from the P-1000. Figure 9c images showed bigger and non-round cavities when compared to Fig. 9b, d. In order to compare the protein integration in the sample, a micrograph was taken from the top of the surface. Figure 9d shows a rough surface, whose concentration corresponds to 250 ppm + CA-V-1B sample, and its porosity is better defined than Fig. 9c, which corresponds to the 2000 ppm + CA-V-1B sample. In that image, a smooth area was presented; its pores are shown in a range of 0.201–8.30 m which represents the largest porosity size dispersion.

**Table 3:** Pore sizes of CA-cellulose viscopearl membranes

Sample T/K 303	*k*_*id*1_ (mg mL^−1^ min^-1/2^)	*C*_1_	*k*_*id*2_ (mg mL^−1^ min^-1/2^)	*C*_2_	*k*_id3_ (mg mL^−1^ min^-1/2^)	$C_{3 }$
CA-V-1A	121.58	22.424	97.403	107.4	0.2527	392.05
CA-V-1B	116.73	236.69	7.5577	483.1	0.1059	507.78
A-V-1A	106.26	44.704	8.6374	258.69	0.1059	285.22
CA-V-2B	99.72	271.28	33.08	401.37	0.399	498.67
C-V-1B	95.967	2.4077	12.956	186.03	0.2118	225.45
CA	97.112	25.179	7.5577	222.1	0.4645	244.4

Table 4 depicts the EDS analysis results in wt%. This test proved that the major constituents for the CA-V-1B, P, and N-P were C and O. The Nitrogen content is included in order to determine the presence of Myoglobin in the samples.

**Table 4:** Energy-dispersive X-ray spectroscopy (EDS) analysis results

Material	Pore size (*µ*m)
CA-V-1B	0.19–0.50
CA-V-1A	0.98–3.34
CA-V-2B	0.75–2.85
C-V-1B	1.64–9.61
C-A	0.31–2.66
P-1000	0.98–5.41
N-P	1.40–6.73
P-250	1.20–6.95
P-2000	0.20–8.30

Calcium was detected in the analyzed zones and the composition of the CA-cellulose viscopearl matrix id referred where only carbon is found. Also, one important matter on doing this type of test was to prove the presence of Calcium in the matrix, which impacts in properties. Furthermore, P sample was characterized with the detection of N which confirms presence of protein during the synthesis. N-P sample was taken after washing the sample for seven times with distilled water; however, no detection of N_2_ was found which suggests that this step washes the protein completely off the matrix. In general, it can be said that all the samples presented an intercalated dispersion of calcium ions and the presence of nitrogen in the samples as supported by the micrographics already described above.

#### Tensile testing

To compare mechanical properties of samples, tests were performed in an INSTRON 3365 tensile test machine (Norwood, MA, USA) at a strain rate of 6 mm/min in accordance to ASTM 882 [73]. Tensile properties were measured on 27 rectangular specimens with a length of 10 mm, a width of 5 mm and a thickness of 1 mm. Values reported represent average from five measurements and typical stress–strain curves were selected for presentation in the graphs.

**Fig. 10:**
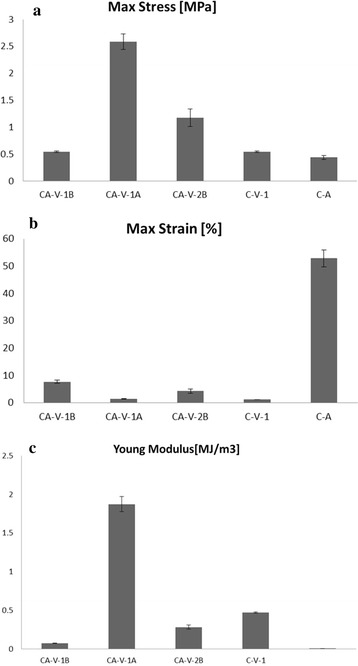
**a** Maximum stresses for all samples in MPa; **b** maximum percentage of strain at which samples; **c** Young modulus for all samples in MJ/m^3^

For the compounds shown in Table 5 and Fig. 10, different formulations were determined based on a prior preparation of materials using Chitosan of low molecular weight (LMW). The results had no mechanical stability and were brittle when handling them. However, one of them could be obtained as a film: the CA-V-1A compound which was then taken into account in the experiments. This will allow evaluation of their behaviour and determine the stress and strain tests, and Young’s modulus. In addition, compounds made of Chitosan of medium molecular weight (MMW) were prepared. The results are compared with those samples obtained from LMW. For this analysis is discarded only for the A-V compound because, as it was mentioned before, it was not possible to prepare the film.

**Table 5:** Mechanical properties of all membrane samples

Material	C (wt%)	O (wt%)	Na (wt%)	Cl (wt%)	N (wt%)	Ca (wt%)
CA-V-1B	39.06	27.42	00.36	19.79	–	13.20
CA-V-1A	33.78	22.99	01.18	24.58	–	17.29
CA-V-2B	39.08	28.71	00.91	19.34	–	11.89
C-V-1B	65.69	33.59	–	00.72	–	–
C-A	23.41	22.84	00.30	26.64	–	26.82
P-1000	58.89	37.86	–	1.56	6.07	1.69
N-P	58.96	37.94	–	1.49	–	00.92
P-250	47.96	23.28	00.15	17.67	4.82	5.90
P-2000	52.01	14.70	00.19	18.19	7.15	7.51

The effect of incorporating porous cellulose beads on mechanical properties of CA-cellulose viscopearls is presented in Table 6. Chitosan–Alginate control film had a tensile strength value of 0.436 MPa. The incorporation of VM into membranes increased tensile strength by 25 % for CA-V-1B and C-V-1B samples, 37 % for CA-V-2B, and 6 times for CA-V-1A. A strong interaction between the Chitosan of MMW, alginate, and VM produced a cross-linker effect, which decreases the free volume and the molecular mobility of the polymer compound. This phenomenon led to a film like structure. Table 6 shows that the tensile strength of blend films increase with increasing VM content up to three times the value of C-A. It also shows that the tensile strength of CA-cellulose viscopearl membranes increase with increasing Chitosan type up to six times higher than that of C-A value and two times higher than that of CA-V-1B and C-V-1B. Despite the fact that products obtained from Chitosan of low molecular weight were expected not to show a good mechanical stability, CA-V-1A shows higher load resistance than the rest of the membranes. Although the sample exhibited the highest load resistance, it was tested to be one of the least deformation resistance materials. Also, it is deduced that VM content is supporting the polymer blending, changing the structure and shape of films and increasing the tensile strength of films accordingly. As a consequence, CA-V-2B sample with the larger amount of viscopearls (0.5 gr) had the second best result in load resistance and presented good deformation, suggesting that the addition of VM in the sample gives further support to the membrane structure. Likewise, compared to CA-V-1B, the increase of viscopearls for CA-V-2B membrane resulted in an increase of 46 % in tensile strength. As expected, the presence of porous cellulose beads and C-A blank material (without porous cellulose beads), improved the Young’s modulus. For samples containing Chitosan of low molecular WEIGHT, the higher Young modulus is presented in CA-V-1A with Alginate and 0.33 gr. The results indicate that 0.5 gr of cellulose beads samples had better mechanical properties than the 0.33 gr sample, as well as higher values of porosity and protein absorption.

**Table 6:** Total energy for compounds involved

Sample	Max stress [MPa]	Max strain [%]	Young modulus [MJ/m3]
CA-V-1B	0.544 ± 0.015	7.615 ± 0.581	0.072 ± 0.003
CA-V-1A	2.587 ± 0.146	1.385 ± 0.138	1.874 ± 0.097
CA-V-2B	1.176 ± 0.165	4.203 ± 0.857	0.282 ± 0.28
C-V-1B	0.544 ± 0.017	1.127 ± 0.016	0.470 ± 0.008
C-A	0.436 ± 0.034	52.781 ± 3.044	0.008 ± 0.000

#### Molecular modelling

Density functional theory (DFT) calculations were carried out for the chitosan, sodium alginate, calcium chloride and acetic acid. For the analysis of reactivity between the substances involved, the possibility of protonation and electrophilic attack was examined by calculating the molecular electrostatic potential at a B3LYP/6-31G(d) level of theory, considering an initial optimization included at the same level. The molecular electron densities and the molecular electrostatic potential surfaces of chitosan, sodium alginate, calcium chloride and acid acetic were determined from the wave functions using CUBE (file with both binary and ASCII formats, which is often used as an input for other graphical visualization) option implemented in Gaussian 09 and visualized using GaussView 5.0 [74] computational software.

An adsorption analysis took place considering the total energy and structural parameters for compounds isolated and in a system of interaction between them, ONIOM calculations were carried out with aid of the Gaussian 09 software package and 6-31G(d) basis set. Additionally, excitation energies from the lowest double energy state were calculated using PM6/6-31G(d) level of theory.

The molecular electrostatic potential has been performed by DFT and ONIOM calculations at B3LYP/6-31G(d) and PM6/6-31G(d) level of theory using PCM solvation model. The adsorption energies and geometrical parameters of acetic acid, sodium alginate solutions, and cellulose have been studied for ground and excited-state geometry to deduce the influence of various substituents as well as the solvent effect on the deformation of molecules.

**Fig. 11:**
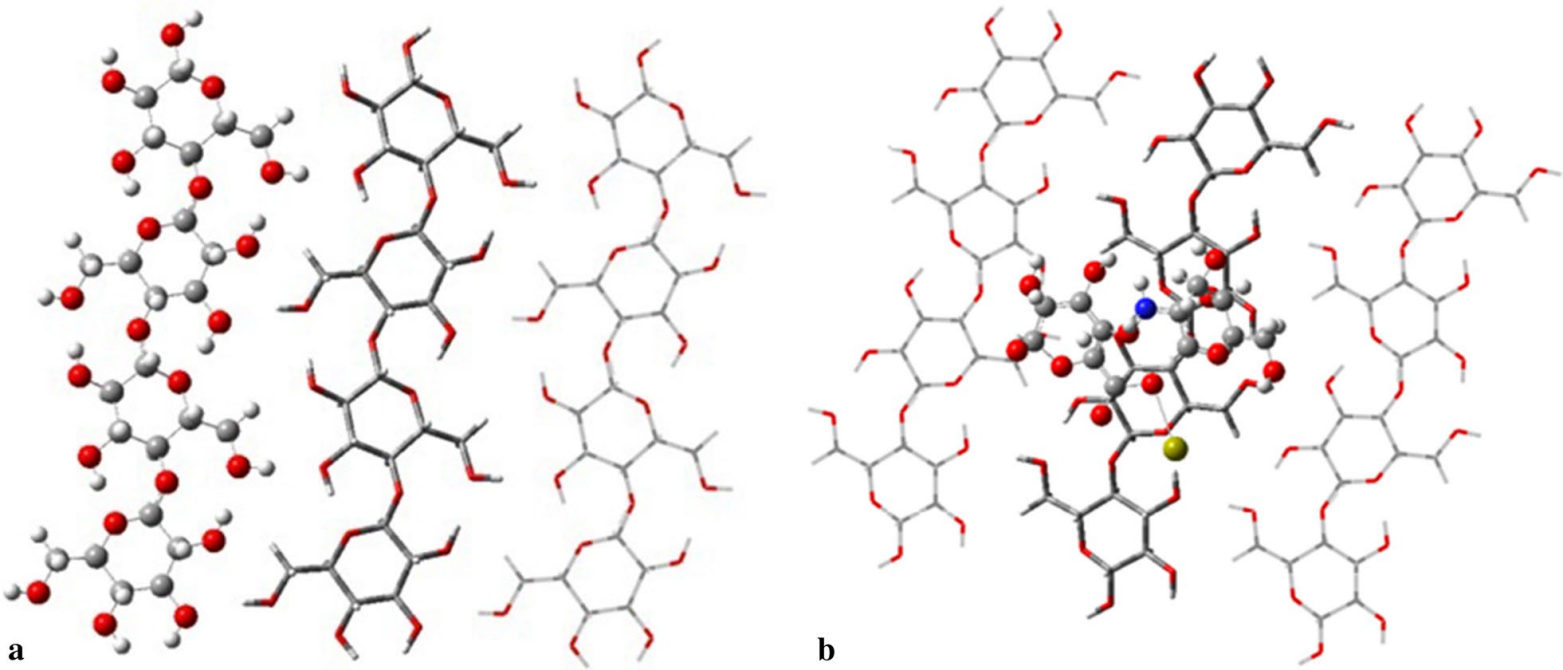
ONIOM’s layers for: **a** Cellulose; **b** Cellulose-Alginate/Chitosan. Corresponding ball and bond type for high, tube for medium and wireframe for low layers

An adsorption analysis took place considering the total energy and structural parameters for compounds isolated and in a system of interaction between them. ONIOM calculations were carried out with aid for the Gaussian 09 software package and 6-31G(d) basis set. Additionally, excitation energies from the lowest double energy state were calculated using PM6/6-31G(d) level of theory. The ONIOM’s layers used for isolated compounds, Cellulose and a complex Chitosan–Alginate, were selected by considering atoms bonded; this is shown in Fig. 11. The results were visualized with GaussView 5.0 software package [74].

### Reactivity

**Fig. 12:**
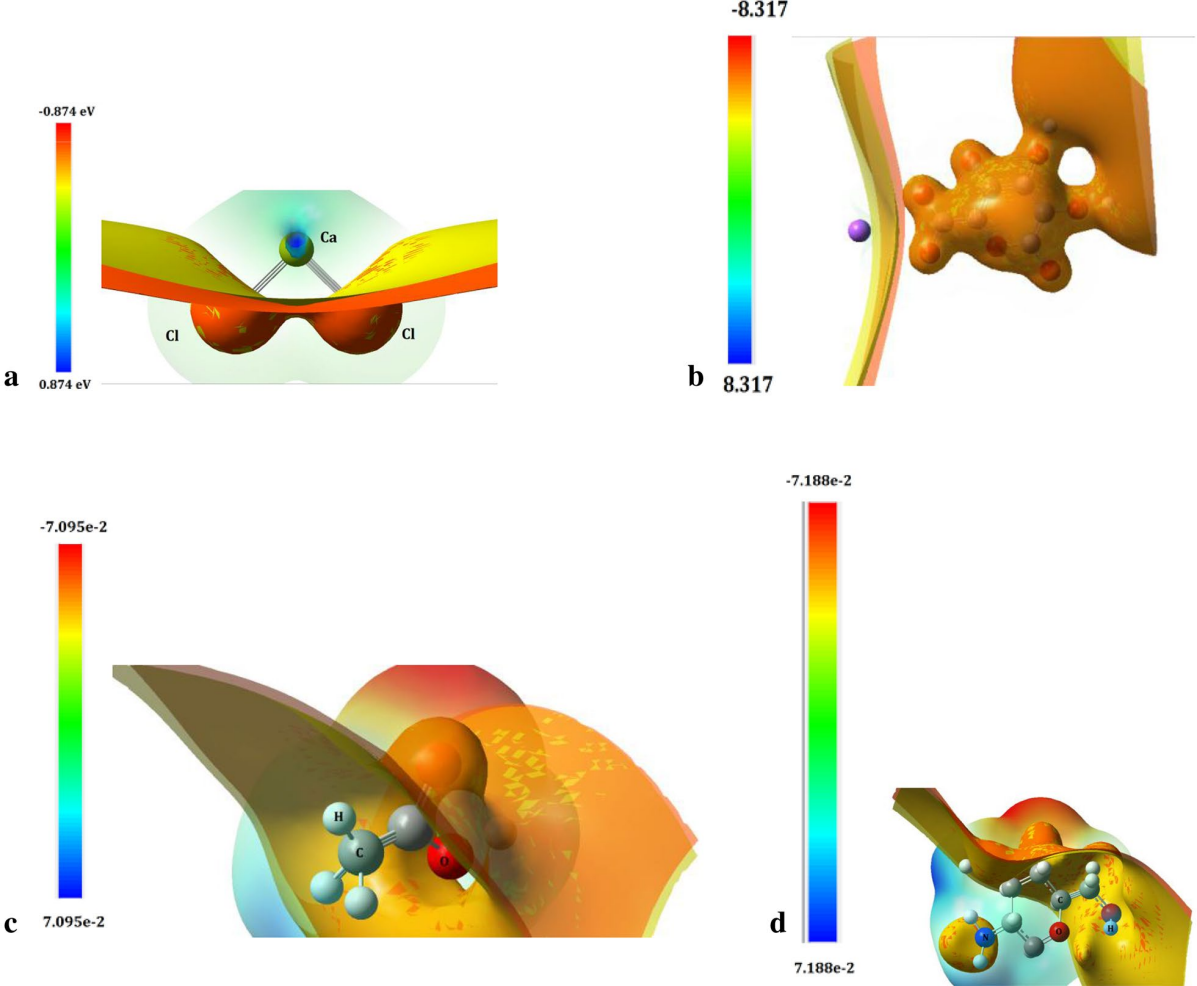
Molecular electrostatic potential computed at a B3LYP/6-31G(d) level of theory with Gaussian 09 and GaussView 5 tools. **a** Calcium chloride; **b** Sodium Alginate; **c** Acetic acid; **d** Chitosan (units are set in eV)

The reactivity process involves an interaction between CaCl_2_ (calcium chloride) and sodium alginate whose potential distributions were computed and are shown in Fig. 12a, b respectively. In them, it is possible to appreciate a negative potential in sodium alginate, −8 eV approximately, surrounding the molecule; for this reason the alginate tends to attract positive ions. In the presence of the high negative potential, the calcium atoms shown in Fig. 12, 0.7 eV approximately were attracted by the alginate, which would result in dissociation of calcium and chlorine atoms. Considering radii of atoms, less than 1 Å for alginate and approximately 2.5 Å for calcium, several alginate´s molecules surround the calcium ion to form a spherical structure. By comparing the potential difference between the alginate and calcium ions, 0.7 and −8 eV, a single alginate molecule will attract several calcium ions to achieve a neutralized system. However, a dilute solution of alginate presents a negative potential a magnitude smaller and therefore less calcium ions attracted.

**Fig. 13:**
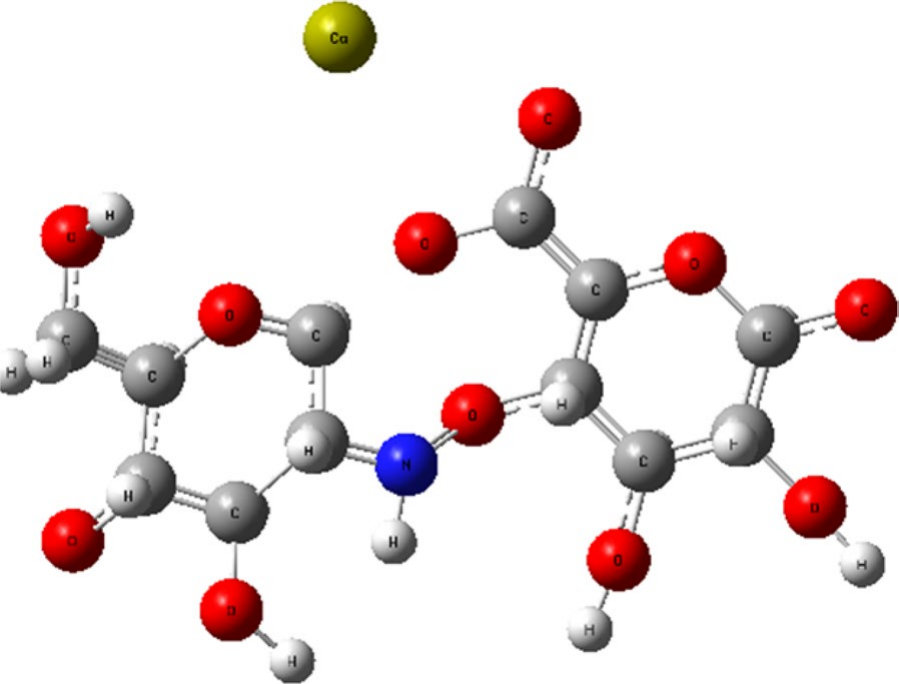
Final structure from Chitosan/Alginate/CaCl_2_/Acetic acid interaction, optimized at a B3LYP/6-31G(d) level of theory

Simultaneously, an interaction between Chitosan and Acetic Acid is established. Considering these molecules, its molecular electrostatic potential (Fig. 12c, d) is obtained individually. In both molecules, the potential has a similar distribution, showing negative regions on one side and positive ones on the other, without incurring any neutral region and all in the order of 1.0 × 10^−3^ eV. This condition can allow proper interaction between the two molecules such that there is a slight attraction between the nitrogen of the Chitosan and the oxygen of the acetic acid to cause an alignment, but no dissociation of either molecule is promoted. Therefore, it is found that the acetic acid presence does not significantly affect the distribution of Chitosan’s potential, so that the suspension remains stable even when carrying out the evaporation of acetic acid. An optimization of the presented molecules was computed, obtaining the total energy for each system, shown in Table 6. According to the potential presented for cases of Chitosan and Sodium Alginate, it is possible to obtain different structures to their interaction, considering the results already discussed, the structure shown in Fig. 13 was obtained. According to this configuration, an adsorption effect was analyzed.

#### Adsorption

An analysis of adsorption energy and structural parameters between an Alginate/Chitosan system and the surface of the cellulose viscopearls was conducted, for which this structure was used by a total of three chains with 12 molecules and the complex Alginate/Chitosan obtained through the analysis of reactivity. A chemical interaction between both compounds does not exist mainly because of treatment with alginate also did not alter viscopearls dimensions [74].

**Fig. 14:**
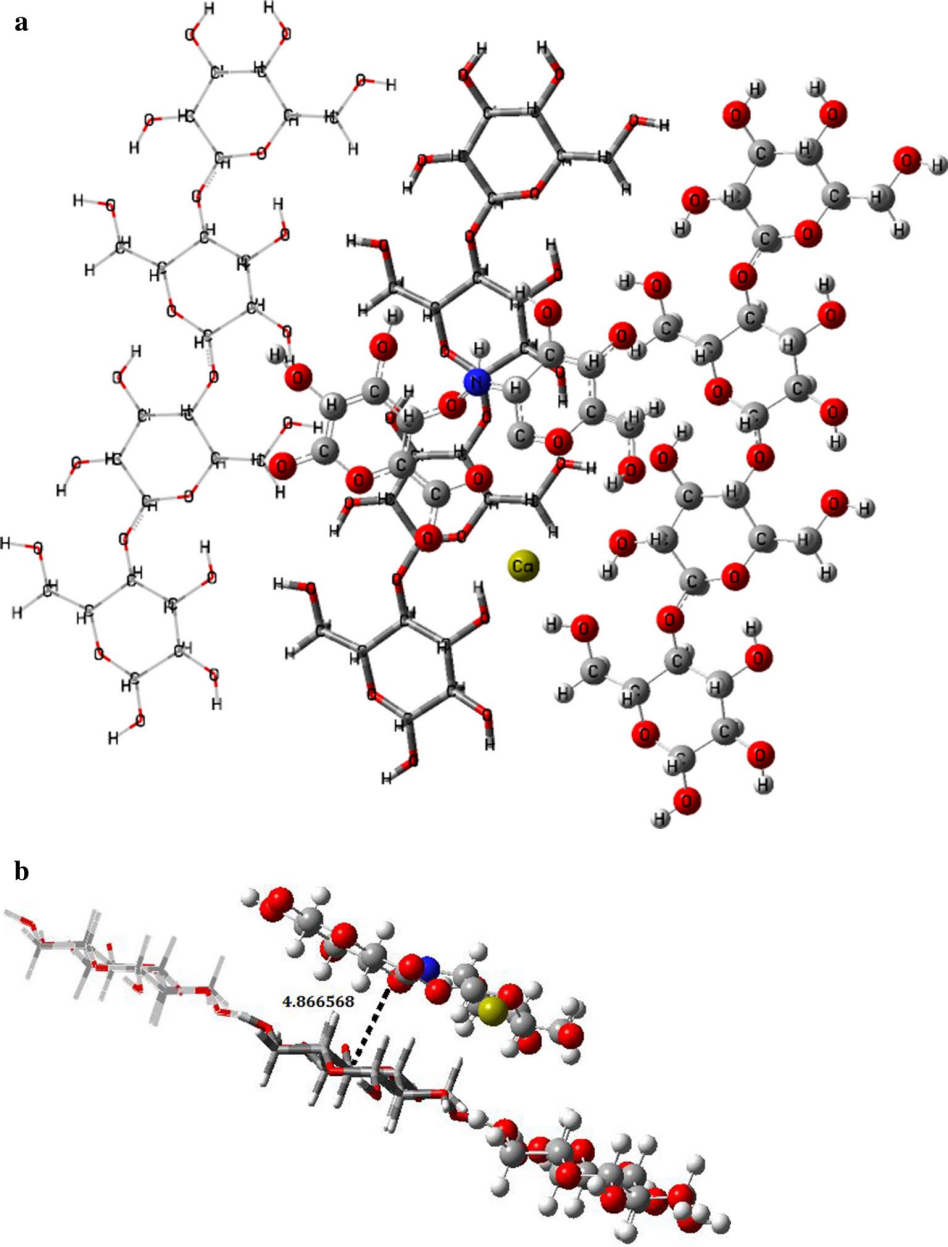
Physisorption structure with ONIOM’s layers: **a** frontal view; **b** transversal view

The possible structure of a cellulose model is fully optimized at PM6/6-31G(d) level of theory at the ground state and then used for a better description of the weak interactions resulting from the physisorption of Alginate/Chitosan complex on the surface of the viscopearls. Then a new optimization of the new system built, Fig. 14a frontal view, b lateral view, was performed, predicting the minimum distance between the adsorbate and the adsorbent with GaussView 5 tools, resulting in 4.8665 Å. It was found that both rings, Alginate and Chitosan, tended to focus around the oxygen of cellulose. Also, the calcium ion is placed in a space free of atoms between cellulose chains.

**Fig. 15:**
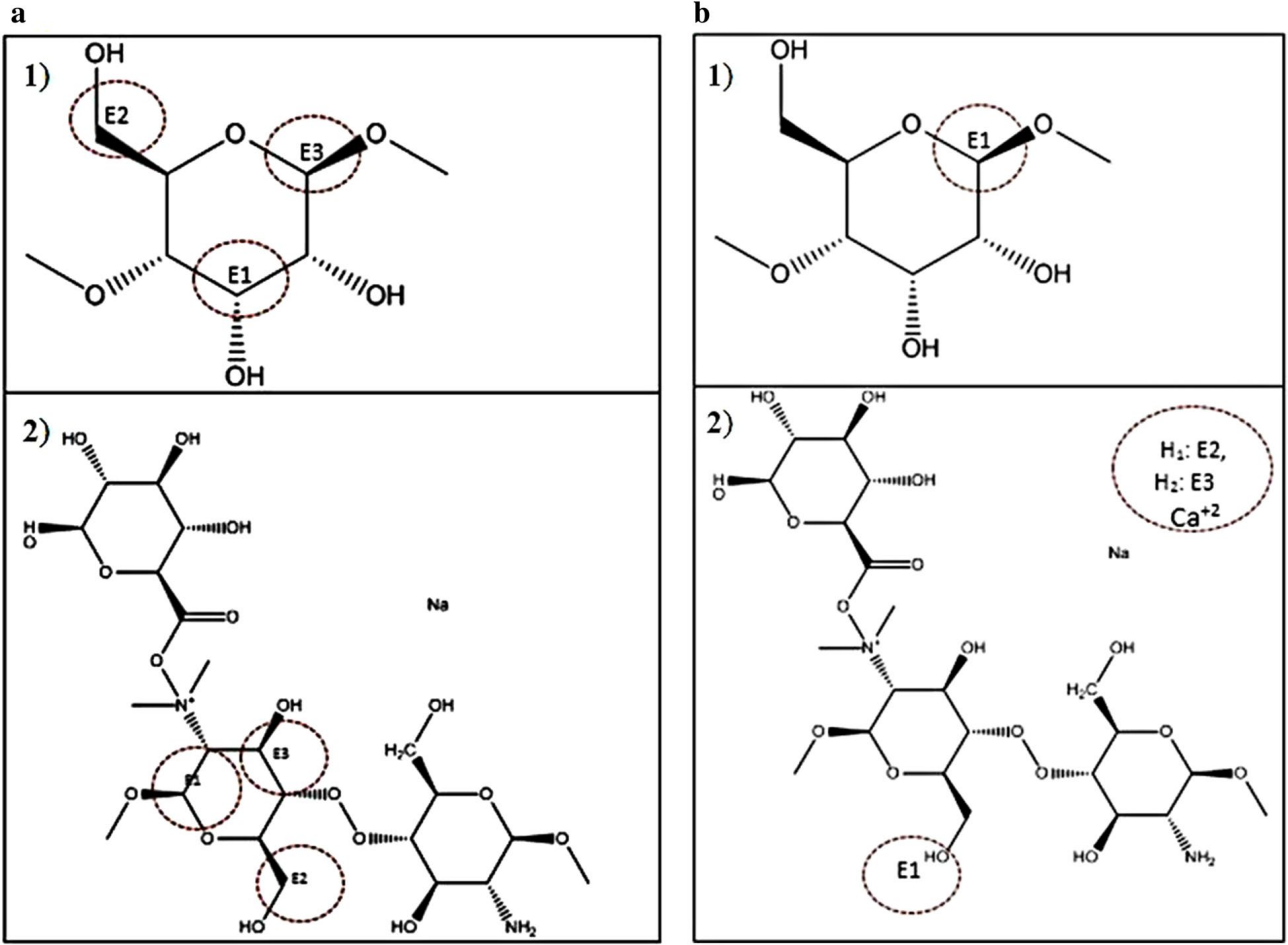
Structure with a linked atom, resulting in a chemisorption effect: **a** configuration 1: *1*. Cellulose and *2*. Alginate/Quitosan; **b** configuration 2: *1*. Cellulose and *2*. Alginate/Quitosan

In the case of chemisorption, there are two optimized configurations. Figure 15a is the configuration done mainly by an interaction of Chitosan; where three bonds appear between the Alginate/Chitosan complex (Fig. 15a.2) and the cellulose surface (Fig. 15a.1). Those arise primarily at the junction between the carbons of the Cellulose and some Hydrogen atoms of Chitosan. Calcium ion is shown by separate from the principal interaction (see Fig. 15b.2), which creates three bonds with the hydrogen atoms of the -CH_2_- and oxygen from the Cellulose (E1). The bond length between the interacting atoms and their neighboring atoms were computed with GaussView 5 tools for both configurations, with the results shown in Table 7. The same parameters for both systems, Cellulose and Alginate/Chitosan, were analyzed separately and shown in Table 8.

**Table 7:** Bond length of atoms linked in the chemisorption process for configuration 1 and 2

Compounds	Total energy (Hartrees)
(a) Chitosan	−589.977
(b) Sodium alginate	−920.739
(c) Calcium chloride	−1598.036
(d) Acetic acid	−228.801

**Table 8:** Bond length of atoms linked in the chemisorption process for two configurations in isolated systems

Bond number	Bond type	Bond length [Å]	Difference [Å]
Configuration 1			
Cellulose			
Bond 1	C–O	1.4102	0.0117
	C–O	1.5271	0.0002
Bond 2	C–O	1.4107	0.0000
	C–H	1.1149	0.0000
	C–C	1.5364	0.0204
Bond 3	C–O	1.4110	0.0124
	C–C	1.5380	0.0106
	C–C	1.5370	0.0006
Alginate/Chitosan			
Bond 1	C=C	1.3300	0.0252
	C–O	1.3300	0.0177
	C–O	1.3297	0.0083
Bond 2	O–H	1.1160	0.0111
	–C	1.3299	0.0092
Bond 3	C–O	1.3300	0.0052
	C–H	0.9748	0.3035
	C=C	1.3297	0.0102
	C=C	1.3299	0.0097
Configuration 2			
Cellulose			
Bond 1	C–O	1.4110	0.0003
	C–C	1.5380	0.0001
	C–C	1.5366	0.0020
Bond Ca 1	H–C	1.1152	0.0000
Bond Ca 2	H–C	1.1152	0.0000
Bond Ca 3	O–C	1.4316	0.0038
	O–C	1.4043	0.0136
Alginate/Chitosan			
Bond 1	O–H–C	1.1168	0.0001

The adsorption energies in both effects, physisorption and chemisorption, considering both configurations, were computed from total energy for each system [75], at first in an isolated form, and then considering the presence of complex Alginate/Chitosan near Cellulose; the results are summarized in Table 9.

**Table 9:** Total and adsorption energies for both configuration in chemisorption effect and structure in physisorption effect computed at a PM6/6-31G(d) level of theory

Bond number	Bond type	Bond length [Å]
Cellulose		
Configuration 1		
Bond 1	C–O	1.4220
	C–O	1.5268
Bond 2	C–O	1.4108
	C–H	1.1149
	C–C	1.5568
Bond 3	C–O	1.4235
	C–C	1.5487
	C–C	1.5364
Configuration 2		
Bond 1	C–O	1.4114
	C–C	1.5381
	C–C	1.5387
Bond Ca 1	H–C	1.1152
Bond Ca 2	H–C	1.1152
Bond Ca 3	O–C	1.4278
	O–C	1.4179
Alginate/Chitosan		
Configuration 1		
Bond 1	C=C	1.3047
	C–O	1.3478
	C–O	1.3214
Bond 2	O–H	1.1049
	–C	1.3207
Bond 3	C–O	1.3247
	C–H	1.2783
	C=C	1.3400
	C=C	1.3397
Configuration 2		
Bond 1	O–H–C	1.1169

The interaction achieved in the different mixture of substances, shown in Fig. 12 (see “Reactivity” section), results in a relatively stable structure with energy of 1.5118 Hartrees. Chitosan and Alginate tend to form a circular configuration around calcium ions, which come from a dissociation of calcium chloride. The Sodium ion is replaced by a calcium one. This new compound interacts with a cellulose surface resulting in chemisorption and physisorption effects, with a minimum distance of 4.8665 Å between each other in physisorption case (Fig. 14b) (see “Adsorption” section). Comparing the two configurations found in the chemisorption effect, Configuration 2 is more stable due to strong bonds from the calcium ion; the adsorption energy obtained was −0.7791 Hartrees, compared with −0.961 Hartrees from Configuration 1. This last structure had an invasive presence due to a range change for the length of the cellulose bonds between 3 × 10^−1^ and 3 × 10^−6^ Å, finding the nearest one at 3 × 10 ^−1^ Å, while on the other side, a length bond change of 1 × 10^−4^ Å exists in Configuration 2. In accordance to these reasons, Configuration 2 was considered the most probable structure; nevertheless, it depends strongly on the initial position in which the complex Alginate/Chitosan arrives to cellulose surface.

Therefore, computational data could suggest that the mix (blend) of CA-cellulose viscopearls agree with the experimental data of protein adsorption. Since adsorption experiments also prove a favorable mechanism for physisorption.

## Methods

### Materials

#### Generals

Cellulose beads (Viscopearl-A) were obtained from Rengo, Japan. Chitosan of low molecular weight (LMW) (viscosity: 20–300 cP), Chitosan medium molecular weight (MMW) (viscosity: 200–800 cP), calcium chloride (reagent plus ≥ 93 %), Acetic acid (pure reagent ≥ 99 %), Myoglobin Protein lyophilized powder from equine heart ≥90 % essentially salt-free, Alginic acid sodium salt from brown algae (medium viscosity). All chemicals used in this study were analytical grade, provided by Sigma Aldrich and used without further purification.

#### Porous cellulose beads (Viscopearl-mini^®^)

A certain type of porous cellulose beads were used for this research. Viscopearl-mini^®^ (VP) or porous cellulose beads obtained from Rengo, Japan with high chemical stability, porosity: <0.01 mm, and range size in diameter: 0.4–0.7 mm [76].

#### Preparation of Chitosan Alginate (CA)-cellulose viscopearl

The preparation process for CA-cellulose viscopearl membranes was carried out by mixing the matrix components according to the formulations shown in Table 1. All solutions were first prepared at room temperature ~30 °C. Alginate solution was prepared following Masalova et al. [77] procedure and two types of Chitosan solution were formulated according to Guo et al. [78], one of them was made from Chitosan of low molecular weight and the other one from medium molecular weight Chitosan.

For each compound, the total blending volume was as much as 6 mL, in which 0.33 or 0.50 gr of Viscopearls-A were added according to each formulation. Then, Alginate solution (previously prepared) was poured in with porous cellulose beads into a petri dish and left overnight. After that, the Chitosan solution was added into the mixture and left for 24 h to dry and to form a thin film which was then stored in a dry environment.

The amount added of Alginate and Chitosan solutions were set at specific concentrations according to Table 10 for all compounds. Finally, the system was kinetically and mathematically analyzed to understand the interactions between the matrix and the different proposed systems.

**Table 10:** Nomenclature for sample synthesized for each formulation

Compounds	Total energy (Hartrees)	Adsorption energy (Hartrees)
Cellulose	−4.0969	–
Alginate/Chitosan	1.5118	–
Chem. configuration 1	−1.6238	−0.961
Chem. configuration 2	−1.8059	−0.7791
Physisorption	−2.7281	0.1431

#### Sample preparation

For all six samples, the solution was stirred manually at 30 °C until a homogenous mixture was attained. The amount of Sodium Alginate solution within the polymeric matrix was kept constant at 3.15 mL in the samples preparation. After the reaction was completed, the different samples were left resting for 1 week to get the diluent to evaporate as much as possible. Afterwards, the prepared materials were press-compressed at 100 °C and 15 MPa for 5 min, followed by cooling at room temperature. Finally, samples were shaped into a desired size for further measurements. Codes names for each formulation sample are listed in Table 10.

#### Adsorption experiments

Batch adsorption studies were conducted to investigate the adsorption behaviour of the CA-cellulose viscopearl membranes. Adsorption experiments were carried out in a 20 mL screw cap tube container with Myoglobin from Horse Heart (MHH) solution containing different CA-cellulose viscopearl samples to study the effects of various contact times (see Table 10).

The different samples were tested using 0.25 g of CA-V-1B, A-V, CA-V-1A, CA-V-2B, C-V-1B and C-A with 1000 mg/L of MHH. To evaluate the effect of initial MHH solution concentration of 500 and 1000 mg/L, different compound samples (CA-V-1B, A-V, CA-V-1A, CA-V-2B, C-V-1B, C-A) were used. All mixtures were agitated manually at 30 °C where contact time varied on a range of 0–30 min. The mixture was then centrifuged and the absorbance of the supernatant was recorded using Shimadzu UV-2500 spectrophotometer (Shimadzu Corp., Kyoto, Japan) using quartz cuvettes with 10 mm path lengths.

All the experiments were performed in triplicate. After the equilibrium, the final concentration *C*_t_ was measured. The percentage removals of MHH solution adsorbed on the CA-cellulose viscopearl membranes, Adsorbed ratio (%), was calculated using the Eq. 8.

8 $${\text{Adsorbed ratio }}({\text{\% }}) = \left( {(C_{0} - C_{\text{t}} )/C_{0} } \right) \times 100$$

where C_0_ and C_t_, are the initial, at time t, and MHH concentration in solution (mg/L), respectively.

Equilibrium adsorption capacity *q*_*e*_(mg/g) was calculated using the Eq. 9

9 $$q_{e} = \left( {C_{0 } - C_{e} } \right)V/M$$

where *V* is the volume of solution (L), and *M* is the mass of the adsorbent (g). The equilibrium data were analyzed using the Langmuir and Freundlich isotherms, and characteristic parameters for the isotherm were determined.

## Conclusions

Chitosan–Alginate membranes containing porous cellulose beads with a homogenous internal structure, as showed by SEM, were successfully prepared from biopolymer blending between the Chitosan–Alginate.

Different morphologies were obtained depending on the formulation system used to incorporate the cellulose viscopearls in order to build the biopolymer membranes. FTIR spectra analysis turned out to be a reliable characterization technique to verify if the principal components stayed in the matrix. NMR in a solid state characterization also helped to determine, from a molecular perspective, the existence of all compounds in the polymer matrix.

To improve the adsorption capacity and mechanical structure of said biopolymer blendings between the Chitosan–Alginate (matrix), a physical interaction between the components is desirable.

Using computational chemistry optimization of the present molecules, the total energy for each system was computed. The interactions achieved in the blending carried out a final matrix compound owning the most stable energy structure; physisorption being the most suitable mechanism of protein interaction.

Tensile tests showed the increase of the amount of cellulose viscopearls was not proportional to the tensile strength. The lesser the cellulose viscopearls were added, the better was the performance found in membranes. This is confirmed their support role on preserving membranes shape, a behavior not observed in the blank sample (Chitosan–Alginate). Finally, the Chitosan–Alginate membrane could not be used to adsorb the protein by itself as the film is brittle and mechanically unstable. Also the prepared blending with cellulose viscopearls could be handled with a sufficient mechanical strength to endure the addressed manipulations and applicability.
